# Implementation of the Swedish Fracture Register

**DOI:** 10.1007/s00113-018-0538-z

**Published:** 2018-09-03

**Authors:** David Wennergren, Michael Möller

**Affiliations:** Department of Orthopaedics, Sahlgrenska University Hospital Gothenburg/Mölndal, SE-431 80 Mölndal, Sweden

**Keywords:** Fractures, bone, Register, Epidemiology, Patient-reported outcome measures, Health care evaluation mechanisms, Knochenfrakturen, Register, Epidemiologie, Beurteilung von „patient-reported outcomes“, Verfahren zur Evaluation der Gesundheitsversorgung

## Abstract

Large financial resources are needed to treat fractures. Surprisingly little is, however, known about actual numbers, treatment methods or outcomes. A large population-based observational study can add valuable knowledge, especially if patient-reported results are included. There is no previous national fracture register with prospectively collected data on fractures of all types, treated surgically as well as non-surgically. With the implementation of the Swedish Fracture Register (SFR), we have shown that this is possible. More than 285,000 fractures have been registered. The database is increasing at a rate of 70,000 fractures a year, i. e. one fracture every 7 min. The aim of this article is to describe the first seven years in the history of the SFR, with opportunities for the future as well as limitations.

## Zusammenfassung

Für die Behandlung von Frakturen müssen hohe finanzielle Mittel aufgewendet werden. Überraschend wenig ist jedoch über konkrete Zahlen sowie Behandlungsverfahren und -ergebnisse bekannt. Eine große bevölkerungsbasierte Beobachtungsstudie kann wertvolles Wissen generieren, insbesondere wenn Ergebnisse, die die Patienten selbst berichten („patient-reported outcomes“), einbezogen werden. Bislang gab es kein nationales Frakturregister mit prospektiver Datenerhebung zu operativ oder auch nichtoperativ behandelten Frakturen beliebigen Typs. Die Implementierung des Schwedischen Frakturregisters (SFR) zeigt nun, dass ein solches Projekt machbar ist. Über 285.000 Frakturen sind bereits registriert. Die Datenbank wächst um 70.000 Frakturen pro Jahr, das heißt um eine Fraktur alle 7 min. Im vorliegenden Beitrag werden die ersten sieben Jahre des SFR dargestellt. Dabei wird auch auf Zukunftschancen und Limitationen eingegangen.

## Swedish national quality registers

National quality registers (NQR) have a long history in the Swedish health-care system. The pioneers were the arthroplasty registers for knees and hips, which date back to the 1970s [9, 10]. Since then, approximately 100 registers with national coverage have been implemented and 14 of them contain data on orthopaedics and orthopaedic trauma [3]. The data are used for research and development to achieve best practice. Awareness of the huge potential of the collected data increased about 10 years ago. Several reports found that increased funding and the increased use of NQRs would be beneficial for the health-care system and most probably cost effective. National funding therefore increased dramatically during a project period from 2012–2016. Many new NQRs were funded and most of them have received annual funding from the Swedish Association of Local Authorities and Regions (SALAR), which is the almost exclusive health-care provider in the country. Currently, the project is under evaluation and the funding has decreased. For the near future, only NQRs that fulfil strict criteria will receive funding. Many NQRs including the Swedish Fracture Register (SFR) find themselves in a difficult economic situation that will obstruct further development and the potential for survival as an NQR is doubtful.

## Start of the Swedish Fracture Register

The creation of the SFR and its early development have been described in a previous publication [12]. The vision of a register for fracture treatment dates back to the mid-1990s. In 2009, these ideas were presented as a pilot model by orthopaedic surgeons at Sahlgrenska University Hospital (SUH), Gothenburg. We were then invited to build the necessary application for data collection by the recently created Centre for Registers in the Western Region of Sweden (Region Västra Götaland, Gothenburg). The structure of the registration platform had already been designed and the idea had been discussed in professional regional and national meetings over the past years.

Entering several fractures on one occasion and further injuries later for the same patient possible

The web-based platform was created to match the needs outlined by the orthopaedic surgeons who invented the concept and data collection was able to begin in January 2011 at SUH. A timeline with opportunities to enter several fractures on one injury occasion and further injury occasions later on for the same patient was the cornerstone of the creation of the registration modules. Data entry is fully web based and performed by the attending physician. By April 2012, more departments had been invited to join the SFR and the national implementation process began.

The SFR has been further developed since the start in 2011. The decision was taken at an early stage to implement and develop the register simultaneously. Since 2015, we regard the SFR as being fully developed with a need for only minor changes in the future.

The SFR is currently being run by a director and a co-ordinator. Only the co-ordinator is employed and funded by the SFR. A steering committee consisting of 20 members includes many orthopaedic surgeons from participating departments and representatives of nurses, physiotherapists and the academic part of the orthopaedic profession as well. The associations for spinal surgery, paediatric orthopaedics and so on are represented on the steering committee. An executive group consisting of the director, the co-ordinator and three individuals from the steering committee runs the SFR on a daily basis. A research board decides on study issues, such as the release of data for researchers that apply for it.

## Implementation

From the beginning, the creators of the SFR have firmly believed that the support of the orthopaedic profession is essential for the SFR to become a success and gain wide acceptance. The NQRs in Sweden have all been started by individual professionals, are all based on a professional need for a register and are still run by professionals with economic support from SALAR.

The idea of an NQR for fracture treatment has to be based on a common perception of the need and benefits for both the profession and the patients. Because of the large number of fractures that are registered nationwide each and every day, it is obvious that registration will increase the work load, even if a single registration only takes 2–3 min. It was essential that the creators and the people involved in the implementation of a register of this kind were all fully active surgeons, well known to the orthopaedic community due to their long-term involvement in teaching, research and national orthopaedic associations. In the Swedish setting, an atmosphere of discussion and consensus is common at workplaces and the hierarchical structure is not as obvious. The role of head of department is more that of presenting arguments explaining why work on registrations is necessary, for example. The head of a department needs to support the register and demand compliance, but the result will not be successful if the idea of a fracture register is introduced from the top down. An NQR has to be useful to individual surgeons, their bosses and the authorities in the health-care system.

Based on the experience acquired from previously started NQRs, it has been estimated that the implementation process for the SFR will take approximately 10 years until full nationwide coverage is achieved. Each year, letters containing information and an invitation to join the SFR have been sent to all heads of Orthopaedic and Trauma Departments in Sweden. This information has been followed by the distribution of an annual report and by presentations and demonstrations of the registration platform at regional and national meetings.

Because the decision to join the SFR and to be a part of this large prospective observational study is not mandatory, we have chosen not to approach the departments in ways other than communication, as mentioned above. Each year, the list of participating departments has grown longer and it is presented in all reports and meetings. Even though mandatory participation would speed up the process, voluntary participation and the running of the register by professionals and not the authorities are felt to be of greater value.

When a department decides to participate, a start-up process usually lasting 3–4 months has been initiated. The SFR provides the hospital with detailed information material and responsible surgeons and secretaries are selected. Their task is to create rules and practical logistics to make it possible to run the SFR at their hospital. Some parts are identical at all departments, but others differ, due to the size of the department, local traditions and so on. After a period of preparation, the Director of the SFR or some other person on the executive board of the SFR pays a 1-day visit to the department. The programme begins with a detailed overview of logistics with the responsible group at the department, including the head of department, and ends with a presentation of the SFR to all available surgeons. The register co-ordinator maintains continuous contact with the secretaries at the departments. The main tasks are adding new users and providing support for the distribution of the questionnaires for patient-reported outcome measurements. An annual meeting is held in January at which surgeons and secretaries from all the participating departments gather for a 2-day meeting with the steering committee, the executive board and the director and co-ordinator.

## Evolution of the register structure

Currently, data on all the extremities, pelvic, spinal, clavicular, hand and foot fractures in adults can be entered in the register. In paediatric patients, fractures of the long bones can be entered. The inclusion criterion is a fracture visible on any modality such as plain radiographs, computed tomography (CT) scans or magnetic resonance images (MRIs). A personal identity number in Sweden is also required and only fractures that have occurred in Sweden are registered. Periprosthetic fractures and fractures close to implants are registered and classified according to the Unified Classification System (UCS; [2]). Specific fracture types, such as pathological, atypical and stress fractures, are also registered.

Data entry is fully web based and includes the registration of the date, cause of injury, low- or high-energy type, fracture classification and treatment and is performed by the attending physician. In the well-functioning departments, a registration rate of over 90% at the A&E in real time has been achieved, even during busy working days in the winter, with a large number of fractures due to falls on ice. Subsequent treatments, new injuries and new fractures can be added to the individual time line.

Pictograms with drawings of fracture types and a user-friendly interface allows for registration rapid

**Fig. 1:**
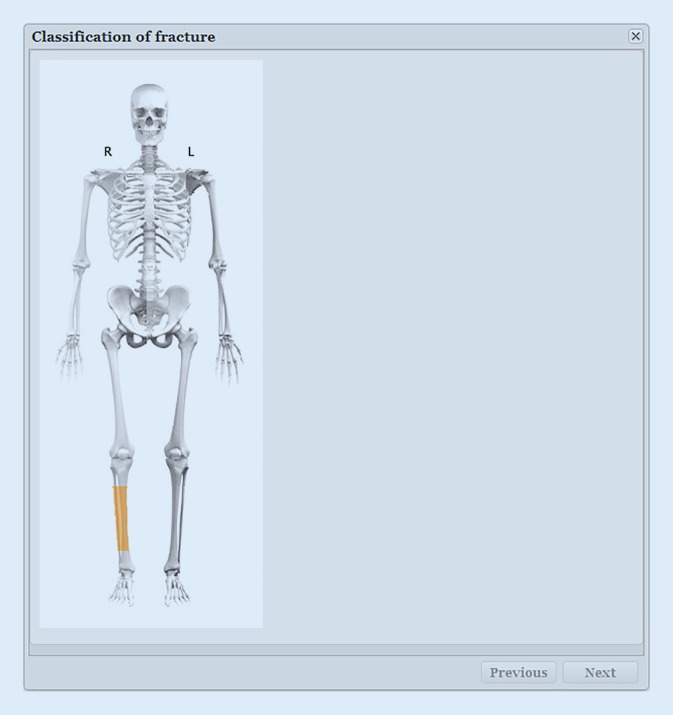
Classification of a fracture starts with choosing the appropriate segment of the injured bone [12]

**Fig. 2:**
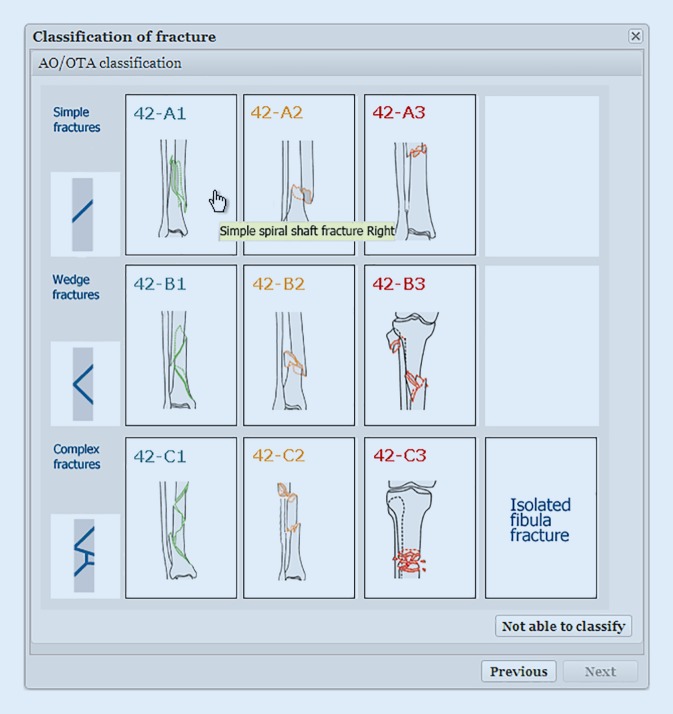
After selection of side and location the classification window appears and the appropriate fracture type is chosen [12]

The registration process has been described in detail in a previous publication [12]. The pictograms with drawings of fracture types is essential in the SFR and has become one of the well-recognised cornerstones (Figs. 1 and 2). A user-friendly interface has been the goal, together with a limited number of variables, which makes the registration process rapid.

## Patient-reported outcome measurements

In 2011 when the SFR was started and when it received its funding from 2012, the creators and the funding authorities felt that patient-reported outcome measurements (PROMS) should be collected. The value of an NQR most probably increases dramatically when the opinions of the patients are collected. After registration in the SFR, all adult patients with a fracture are offered the chance to answer a questionnaire with the EQ-5D-3L, the Short Musculoskeletal Function Assessment (SMFA) and a few additional questions relating to health status and level of functioning before the fracture occurred. The patients that fill in the questionnaires are sent identical questionnaires one year later. Together with the re-operation rate, the PROM results are the main outcome variable in the SFR.

For legal and logistical reasons, the SFR still uses standard mail, even though the ambition for the future is to be able to collect the same information using other more modern techniques.

The response rate is much lower for fracture patients than for patients that undergo elective surgery, such as arthroplasties. A fracture patient might have had a fracture of only minor significance. Several patients are cognitively impaired and many are old with problems relating to sight, motivation and other complicating factors. In the younger patient group that sustains fractures, there are also subgroups of drug addicts, criminal behaviour and other factors that limit the expected number of responses. However, if there is no systematic bias between responders and non-responders, a limited response rate is also useful when attempting to understand and analyse outcome after fractures. In studies performed in the SFR, no systematic bias has been detected [5].

The results of PROMs are available to all users of the SFR, together with the response rates. The response rates are somewhat difficult to evaluate because the actual percentage of the whole fracture population that has received a questionnaire after fracture is not known. We do know the number of patients registered in the SFR, but we do not know whether all patients have actually received the PROM questionnaires. Having said this, the actual response rate of approximately 50% after the fracture and 35% after one year in the SFR is still useful and unique.

More than 60,000 PROMs with answers from both occasions currently included in the SFR database

More than 60,000 PROMs with answers from both occasions are currently included in the SFR database. PROM-based results were recently published on the website as a quality indicator comparing the performance between the departments in Sweden. The outcome after surgically and non-surgically treated ankle and wrist fractures is currently used. It is challenging to interpret PROM data and it will take time to understand what the patients are telling us about their level of function after our treatment of their fractures. We must nonetheless try. There is no better way.

## Registration of re-operations

Re-operation rates are the main surgeon-reported outcome registered in the SFR. All treatments given are registered. Primary treatments (surgical as well as non-surgical) are distinguished from planned secondary treatments and re-operations. If non-surgical treatment at an early stage is converted to surgical treatment, due, for example, to increasing dislocation at an early X‑ray check, this sequence is recorded as well.

Re-operations and late operations after failed non-surgical treatment, e. g. mal-union, are regarded as failures because they were not part of the original treatment plan. Re-operations are subgrouped according to the reason for the re-operation.

The achievement of a high completeness level in the registration of re-operations contains several challenges. It is mainly necessary to rely on the surgeons remembering to enter the re-operation in the register. The validation of the completeness of re-operations can be performed by comparing with official health databases, which is done regularly by the arthroplasty registers. This is, however, much more difficult in a fracture setting, due to the large number of possible treatment codes to check against. There are several ongoing studies in the SFR for validating the completeness of re-operations by reading thousands of file notes. When presenting data from a register such as the SFR, it is important to be aware of pitfalls of all kinds and to try to control for them. If, for example, the performance of different departments in a register based on re-operation rates is compared, it might turn out that the department with the poorest figures is in fact the department with the highest completeness of registrations of re-operations.

An NQR in Sweden can, as the SFR does, use a real-time link to a national database for all citizens. When entering the personal identity number, key data on the individual, such as name, address and so on, are returned and the registration can take place. When a Swedish citizen dies, this information automatically appears in the SFR within a few days, enabling straightforward studies of mortality rates in the register without the need for other data sources.

One challenge when studying the outcome after fracture care is that the patient might receive treatment more than once for the fracture. There might also be more than one fracture on the same occasion and the fractures might be treated at different hospitals both in the acute setting and later on. To ensure the best possible way of following the path of the patient, only one entry of baseline data is used, i. e. patient, injury occasion and fracture. Subsequent treatments can be added to the originally registered fracture by surgeons at other hospitals later in the process. This diminishes the risk of double registrations.

## Coverage and completeness

**Fig. 3:**
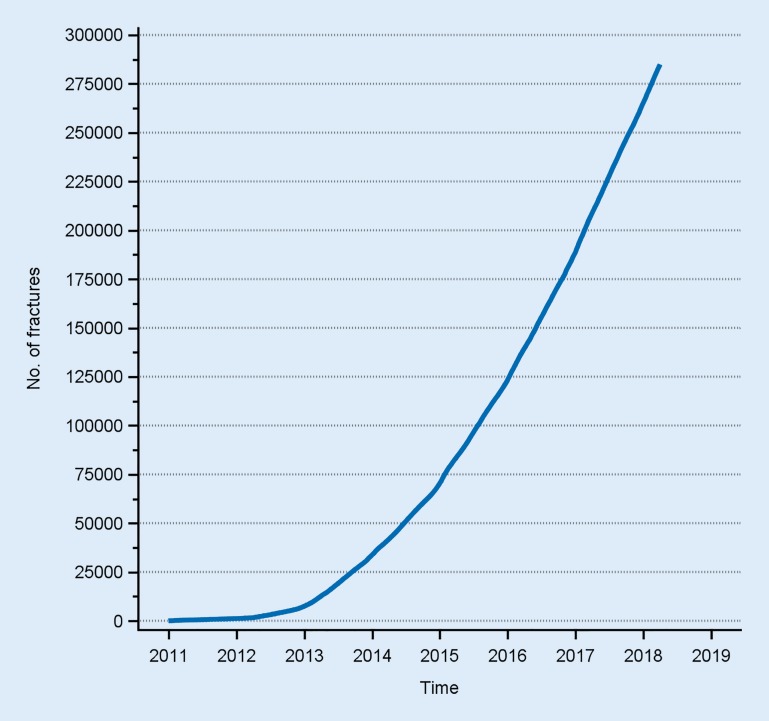
The cumulative frequency of fracture registrations in Swedish Fracture Register (SFR) since the start

The SFR is now functioning effectively and is used as a clinical routine at the majority of the orthopaedic departments in Sweden. From January 2011 to June 2018, more than 285,000 fractures have been entered at 46 Swedish orthopaedic departments (Fig. 3). Approximately 40 of 55 Swedish departments are entering data on a regular basis. A few departments have chosen to start the process of entering data with a limited number of fracture types, e. g. only spinal fractures. The coverage is therefore between 70–85%, depending on the definition that is used. The departments that participate are spread throughout the country and in all regions. There are small hospitals, medium-sized hospitals and the largest hospitals in the country. Almost all the large hospitals with a large number of fractures, including most of the university hospitals, are contributing to the data collection.

Completeness has reached 70–95% for wrist, humerus, ankle and hip fractures in most departments

The completeness of an NQR is essential. The larger the percentage of all fractures that are entered, the higher the level of completeness and the more valuable the data. Comparing registrations in an NQR with the official health databases in Sweden is a well-known problem. Entries are compared on the basis of the ICD codes for diagnosis, which creates several potential sources of error, but it is still the only way for an NQR to estimate its completeness. The SFR has created an algorithm in collaboration with the National Board of Health and Welfare, which runs the analyses of completeness between NQR data and data from the National Patient Register. Data for the last two years show a completeness of 70–95% for fractures to the wrist, humerus, ankle and hip at most of the participating departments. Not surprisingly, the highest figures are obtained for hip fractures, which are a common fracture type treated almost exclusively by surgery, whereas wrist and ankle fractures have a lower completeness level. If a fracture type is generally treated on out-patient visits and non-surgically, as is the case with wrist fractures, it is more difficult to achieve a high completeness level. The completeness data per hospital will be published for the first time in the annual report for 2017.

## Communication

Data that are not analysed and communicated back to the users, health authorities and, in the end, our patients, are of no use. Too many quality registers have collected data without a valid plan for communication with users. In a register like the SFR, the person that enters data is the same person that subsequently wants to see the results and use them in clinical practice. At your own department, you have access to data linked to PINs which enables you actually to follow every single patient. This can be useful and possible for rare fracture types and in special situations. All users at all departments can also see aggregated data and compare the figures and results for their own department with the average in the country and with every other hospital.

Communication on important issues relating to the development of the register takes place by a newsletter distributed three to four times a year by e‑mail to all 2500 users. The web page also serves as an archive for news and information. All ongoing research projects are published with project plans. Each year, an annual report is published both as a booklet and electronically on the website (frakturregistret.se). In this report, many aspects of the SFR are covered using news, developments and a large section on statistics. Different fracture locations have been focal areas in different reports. In the report for 2017, we analyse hip fractures, wrist fractures, elbow fractures, foot fractures, hand fractures, femur fractures in children and spinal fractures.

During the years since the start of the SFR, there have been almost yearly presentations at national meetings, in addition to scientific presentations and poster presentations. As the SFR has grown, interest from outside Sweden has been growing and presentations have also been made at a European Federation of National Associations of Orthopaedics and Traumatology (EFORT) meeting. Groups of interested surgeons from various countries have visited Gothenburg to learn more about the SFR and active collaboration is currently ongoing with the Norwegian fracture register initiative.

## Use of data in clinical practice and for research

In the SFR database, there are currently data on 56,300 wrist fractures, 50,700 femoral fractures, 31,700 humeral fractures and 28,500 ankle fractures. The data can be used for planning, follow-up and changes in clinical practice. When a user is logged into the SFR website, he or she can easily access real-time data. As previously mentioned, the available data can be divided into aggregated data and data specific to one department with the opportunity to identify individual patients. The aggregated data are predefined into 15 modules. In each module, the users can filter using most of the available variables to create customised graphs and figures. For example, the number of fractures per month, year, average and so on can be displayed compared with other departments and filtered in age groups, gender, fracture types and so on. The percentage of a fracture type that is treated surgically is another example of the available data. The percentage of hip and femur fractures operated on within 24 or 36 h from the time of the X‑ray is presented. Re-operation rates, PROM results and PROM response rates can also be displayed.

Identification of osteoporotic fractures may allow start of preventative treatment

Probably the most useful tool in clinical practice when accessing a department’s own data is the search for possible osteoporotic fractures. Finding an osteoporotic fracture is a well-known problem worldwide. Great efforts have been made to find patients with osteoporosis that have sustained a first osteoporotic fracture and offer them the opportunity of investigation with dual-energy X‑ray absorptiometry (DEXA) and, if needed, a proposal for pharmacological and other treatment for osteoporosis to try and prevent the next fracture. In the SFR, a co-ordinator can press the button entitled “Find patients over the age of 50 with a suspected osteoporosis-related fracture”. The database will return the PINs of patients with fractures to the proximal humerus, wrist, hip, pelvis or spine for the period of time chosen.

Many research projects have been started and seven of them have been published during the last 3 years [1, 5, 6, 8, 12–14]. Interest had focused on validation studies and descriptive data. Of these studies, three have evaluated the validity of the classification of fractures in the SFR [6, 13, 14]. In these studies, the gold standard classification for each fracture was defined by senior surgeons. The gold standard classification was then compared with the actual classification in the SFR to evaluate the accuracy of the classification in the SFR. The result was as good as in previous studies evaluating experts classifying fractures, even though the classifications in the SFR were generally made by junior doctors at the A&E. There are many ongoing projects involving evaluations of results, including re-operation rates, PROMs and comparisons of different treatments. In the near future, it will also be possible to organise randomised trials within the SFR, so-called R‑RCTs.

## Difficulties and opportunities

There are many difficulties involved in implementing a nationwide fracture register. The best way to ensure the long-term sustainability and survival of a register is to be aware of the difficulties. Gaining the acceptance of colleagues is the most important at the start. It is of course essential to convince the heads of departments to join the register, as is the funding. If published data are useful when it comes to changing the way we treat fractures in order to improve the situation for our patients, it is more likely that the register will continue to receive funding. One recent problem is whether to change the whole structure of the register to match the new version of the AO/OTA Classification that has been developed [7].

The legislation and the PIN in Sweden make it relatively easy to run a quality register compared with many other countries. The possible benefit of large observational studies of outcome after fractures, including PROMs, is large. This has also been stated by the authors of the PROPHER (a randomized-controlled trial [RCT] on humeral fractures) [11]. They wrote in an appendix to the published study that initiating further RCTs is not appropriate—“The setting up of a national database of these fractures with the systematic and prospective collection of data on epidemiology, management and outcome, including patient-reported outcomes, should be considered” [4]. The SFR fulfils the vision of the authors of one of the most discussed fracture RCTs in recent years.

The creation of a platform on which it is possible to run randomised studies within the register will also utilise the full potential of the register in the near future. It will hopefully also be possible to add registrations of implants into the SFR, using barcode detectors in the operating theatre. The exchange of data between registers in Sweden enables studies of many variables primarily not included in the SFR, e. g. comorbidity. If the profession agrees, it will be possible to register single surgeons. It will also be possible to publish analysed data with comparisons between departments for the public and not only the profession.

### Ethics

The Swedish Fracture Register is approved by the Swedish Data Inspection Board and operates in accordance with Swedish legislation, i. e. the Swedish Personal Data Act, the Swedish Patient Data Act and, since May 2018, General Data Protection Regulation (GDPR). All patients are informed that registration will take place and that they have the right to decline. According to Swedish legislation, NQRs do not require signed consent from the individual registered patient. The benefit of this opt-out system for NQRs in Sweden cannot be overestimated. The research conducted in the SFR has been approved by the Central Ethical Review Board, Gothenburg.

## Practical conclusion

- The creation and implementation of the SFR have shown that it is possible to register both surgically and non-surgically treated fractures nationwide.
- It is possible to obtain patient-reported results regarding function after fracture treatment on a large scale.
- The Swedish legislation and previous experience make large observational studies feasible.
- A fracture register has to be implemented from the bottom up to be sustainable in the long term.
- The involvement of the orthopaedic profession is essential if the register is going to be successful.

## Abbreviations

A&E: Accident and emergency department
AO: Arbeitsgemeinschaft für Osteosynthesefragen
EQ-5D-3L: Euroqol 5 dimensions 3 level
GDPR: General Data Protection Regulation
ICD-10: International Classification of Diseases Tenth Revision
NQR: National Quality Register(s)
OTA: Orthopaedic Trauma Association
PIN: Personal identification number
PROM: Patient-Reported Outcome Measures
SALAR: Swedish Association of Local Authorities and Regions
SFR: Swedish Fracture Register
SMFA: Short Musculoskeletal Function Assessment
SUH: Sahlgrenska University Hospital
